# Excision of the Spleen

**Published:** 1844-10-05

**Authors:** 


### RECORD OF MEDICAL SCIENCE.

### EXCISION OF THE SPLEEN.

M. Berthert of Gray, relates a case of excision of the spleen. An individual received, in a quarrel, a cut with a knife in the left side. Eight days after the accident, M. Berthert, on being called in, found a considerable tumour formed by the spleen, which exhaled a strong smell of putrefaction. He excised the tumour, the surface was methodically dressed for some time, and healed. The patient lived more than thirteen years afterwards, and his digestions were always accomplished with ease, which seems to prove that the spleen is not more necessary to life in man than in the animals from which it has been excised of late by vivisectors. This individual died of pneumonia. Only a very small portion of the spleen, as large as a nut, was found; it was applied on the external parietes of the stomach.—*Lon. Lancet.*

